# Motor Skill Competence Matters in Promoting Physical Activity and Health

**DOI:** 10.1155/2021/9786368

**Published:** 2021-12-24

**Authors:** Zan Gao, Xu Wen, You Fu, Jung Eun Lee, Nan Zeng

**Affiliations:** ^1^School of Kinesiology, The University of Minnesota, 208 Cooke Hall, 1900 University Ave. SE, Minneapolis, MN 55455, USA; ^2^Department of Sports Science, College of Education, Zhejiang University, Hangzhou, China; ^3^University of Nevada at Reno, Reno, NV, USA; ^4^Department of Applied Human Sciences, University of Minnesota, Duluth, MN, USA; ^5^Prevention Research Center, Department of Pediatrics, University of New Mexico Health Sciences Center, 2703 Frontier Ave. NE, Research Incubator Building (RIB) Suite 120 Albuquerque, NM 87131, USA

Children's low physical activity and health-related physical fitness levels may increase their risk of developing chronic diseases such as type II diabetes and hypertension [1, 2]. Participating in daily physical activity regularly is imperative in enhancing children's motor skill competence early in life and health promotion across the lifespan [3, 4]. As known, the development of motor skill competence has been posited to be an important predictor for increased levels of children's health-related physical fitness and physical activity behaviors, as well as improved health outcomes [5–9]. Motor skill competence has been defined as common fundamental motor skills and goal-oriented movements including large muscle groups or the whole body [10]. In general, motor skill competence consists of three major components: locomotor skills (i.e., the ability to move the body through space like running, jumping, and hopping), object control skills (i.e., the ability to move and manipulate objects in space like kicking, throwing, and catching), and stability skills (ability to maintain postural control like balancing, rolling, and stopping) [11].

Cross-sectional studies showed that children's motor skill development is positively linked to their health-related physical fitness, enjoyment, and perceived competence [12, 13]. Longitudinal studies also have shown that children's motor skill competence resulted in sustained levels of physical activity and health-related fitness over time [14]. Thus, the study of correlates and determinants of motor skill competence, physical activity, and sedentary behavior across the lifespan has become an emerging trend in the field, as, increasingly, researchers have conducted investigations in this area of inquiry in the past decade.

In response, we invited investigators to contribute original research articles and review articles that would stimulate continuing efforts to understand the relationships between motor skill competence, physical activity, health-related physical fitness, and health outcomes among various populations. In this special issue, novel findings from seven separate studies from multiple countries are presented. These studies attempted to (1) systematically review the effects of physical activity programs on motor skills and health outcomes in pediatric populations; (2) investigate the effectiveness of physical activity programs on individuals' motor skill competence; (3) provide empirical evidence concerning the relationships among individuals' motor skill competence and physical activity, physical fitness, and academic achievement; (4) test the validity of children's movement skill quotient based upon classroom environment; and (5) offer directions for future research studies in this area of inquiry.

D. J. McDonough et al. [15] synthesized 25 randomized controlled trials (RCTs) examining causal evidence regarding the effects of physical activity interventions on children's motor skill development. They found that 20 RCTs reported significant improvements in motor skill performance as a result of physical activity participation. In detail, 18 studies examined traditional physical activity interventions and 7 studies examined exergaming-based physical activity interventions, 15 and 5 of which observed statistically significant improvements in children's motor skill development, respectively. Of note, some limitations of this review (e.g., heterogeneity of measurement protocols and assessment tools in testing motor skills) are discussed. Thus, the authors advocated for future RCTs to employ a priori power analyses and long-term follow-up measurements, to explore the dose response and moderating relationships between physical activity and motor skill development in childhood, and to utilize homogenous assessment instruments to allow for more rigorous, quantitative syntheses. J. Yin et al. [16], on the other side, reviewed RCT studies that compared high-intensity interval training with moderate-intensity continuous training on health outcomes among children and adolescents. In this meta-analysis of 16 RCTs that included 543 participants published from 2005 to 2019, children and adolescents demonstrated a significantly higher peak VO_2_ level after participating in high-intensity interval training compared to moderate-intensity continuous training. However, no differences were reported between modalities on the outcome variables such as maximum heart rate, fat mass, free fat mass, weight, body mass index (BMI), waist circumference, blood pressure, glycemia, insulinemia, total cholesterol, high-density lipoprotein, low-density lipoprotein, triglycerides, homeostatic model assessment of insulin resistance, hemoglobin A1c, and leptinemia. The authors suggested that more large-scale RCT studies should be conducted to further investigate the effects on young individuals' health outcomes between high-intensity interval training and moderate-intensity continuous training.

To gain empirical evidence, H. Wang et al. [17] tracked young children's motor skill development over time and investigated the determinants from home environments. The Test of Gross Motor Development tool (version 2) was used to assess 268 children's locomotor and object control skills, as well as self-reported behavior and home environments. During the follow-up, children's locomotor and object-control skills continued to grow, with an annual growth rate of 20% and 30%, respectively. Specifically, the scores of 5 locomotor skills and 2 object control skills were significantly higher in the 3–4-year-old group than in the 4–5- and 5–6-year-old groups. Girls' locomotor skills developed at a significantly higher rate than those of boys. Furthermore, 3-year-old boys performed significantly better than girls on object-control skills. Determinants of children's motor skill development in middle-income families include the frequency of playing with friends and the frequency of bicycling, skateboarding, dancing, running, and jumping. Family income, parents' education level, and family activity area were not significantly associated with the improvement rate of motor skills. In addition, more opportunities to play with friends and engage in a variety of sports activities could promote children's motor skill development. Meanwhile, M. Batez et al. [18] examined the relationships between motor competence, physical fitness, and academic achievement in young children. The Körperkoordinationstest Für Kinder and the EUROFIT battery of tests were applied to measure motor competence and physical fitness, respectively, in 130 elementary school children in Serbia. Academic achievement was assessed based on the grade point average (GPA) score. It was found that the GPA scores significantly correlated with almost all motor competence and physical fitness measures. The results of regression indicated that only plate tapping and sit and reach could significantly predict the GPA score. The authors concluded that academic achievement is generally associated with physical fitness and motor competence in children. Plate taping and sit and reach were accounted as the most important predictors for academic achievement. The study may contribute to further understanding of the link between motor competence, physical fitness, and academic achievement.

On the other lifespan spectrum, degenerative changes such as muscle loss, weight gain, and osteoarthritis occur in seniors during the aging process, which increase the risk for falling and cognitive decline among older adults. Thus, actions are needed to prevent and treat degenerative changes in this population. To this end, J. Adamczyk et al. [19] conducted a RCT to examine an exercise-based intervention on older women's physical functioning. A total of 73 participants were recruited and randomly assigned to either the intervention group or the control group. The intervention group was required to participate in a Jaques-Dalcroze eurhythmics exercise program for 12 weeks, twice a week for 45 minutes each session, whereas the control group was asked to maintain regular activity patterns. Dynamic agility was determined by the Timed Up and Go test for both single-task and dual-task as the primary outcome. After 12 weeks of exercise intervention, the authors observed that only the dual-task scores were significantly higher in the intervention group, compared to the control group. The finding supports Jaques-Dalcroze eurhythmics as an effective intervention form to improve physical functioning in women over 65 years of age.

Working with an athletic population, W.-D. Chang et al. [20] examined the relationships between various motor ability tests and sports injury risk among 32 athletes who either played volleyball, basketball, or handball in school sports teams for at least 3 years. The tests were functional movement screen, star excursion balance test, agility T-test, and vertical jump test, and the differences in the test scores were compared between groups with high- and low-injury risk. No significant differences were observed in the functional movement screen, balance test, agility T-test, and vertical jump between high-risk and low-risk groups. Additionally, a series of moderate-to-good correlations were observed for deep squat, inline lunge, hurdle step, rotary stability of functional movement screen, and various components of the balance test. Researchers concluded that junior athletes with a functional movement screen score of less or equal to 14 or a balance test score difference of 4 cm or greater have a higher risk of sport injury, and these moderate correlations between functional movement screen and balance test scores may be due to similar movement patterns required among these tests.

As known, a reliable and valid assessment tool is essential for evaluating children's movement skills in daily physical education environments. J. Chang et al. [21] examined the validity of the Children's Motor Skills Quotient used in the physical education setting. A total of 734 children completed the 14 test items (e.g., jumping, sliding, catching, throwing, crawling, running, bouncing, rolling, and kicking) which were evaluated by six raters. Rasch analysis was used to verify the fitting statistics, project difficulty, and functional differences of the items of the Children's Motor Skills Quotient. The findings showed that the instrument met the assumptions of the Rasch model, including the unidimensionality, local independence, person measure, and item difficulty hierarchy. The instrument also demonstrated adequate interrater reliability and internal consistency. The differential item functioning demonstrated a number of items showing different probabilities across sex and age. Overall, the Children's Motor Skills Quotient seemed to have appropriate testing items with an appropriate rating scale structure for measuring 6-9-year-old children's movement skills in the physical education environment.

## 1. Challenges and Opportunities

**Figure 1:**
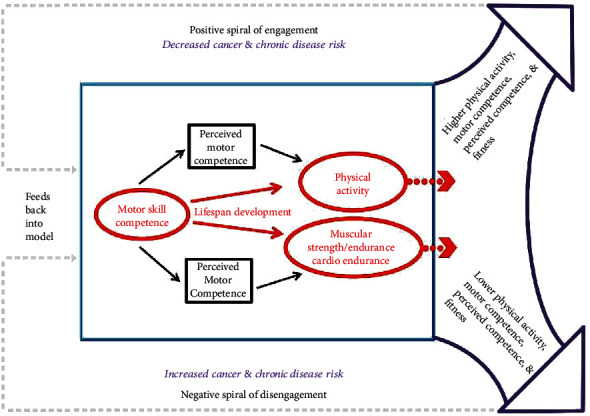
Spiral of engagement (Stodden et al., 2008).

According to Competence Motivation Theory [22], there is a positive relationship between one's behavior and motor skill competence and perceived competence. In particular, successful skill/task mastery (e.g., sports-specific motor skill competence development) will enhance one's perceived competence, which in turn, facilitates other motivated behaviors (e.g., engaging in physical activity) and performance on physical tests (e.g., motor skill competence and fitness testing scores) [5]. Compiling the literature has observed a trend such that many children who have lower physical activity-related perceived competence (e.g., confidence in playing football) avoid physical activity opportunities because (1) they feel less physically competent compared to their peers [23] and (2) they are ashamed/embarrassed to demonstrate low motor skill competence. Since these children have fewer athletic skills and, therefore, less movement opportunities, they will be less motivated to participate in physical activities or sports activities which, compared to their more advanced peers, will be less fun for them [24]. Indeed, cross-sectional and longitudinal studies suggested that the relationships between children's motor skill competence and physical activity, and fitness might be mediated by their perceived competence [25]. However, it is still unclear whether this mediating effect of perceived competence is prevalent among young children who possess high levels of perceived competence and low levels of motor skill competence, concurrently. Furthermore, the maintenance or development of motor skill competence can be achieved by regular participation in physical activity and high levels of physical fitness [25]. However, whether or not perceived competence mediates the effect of physical activity and health-related physical fitness on future motor skill competence has yet to be examined, thereby creating the need for future research on this topic among young children.  Figure 1 shows two feedback loops for the preceding engagement (i.e., negative or positive). In detail, a “positive spiral of engagement” suggests a positive trajectory for physical activity and physical fitness with high motor skill competence, whereas a “negative spiral of engagement” suggests that low motor skill competence may lead to lower levels of perceived competence, thus decreasing physical activity and fitness over time, and, ultimately, poor future motor skill competence in children. Participation in innovative and fun physical activity promotion programs (e.g., active video game programs [5]) may facilitate the development of motor skill competence, promote these recursive relationships, and may even have the potential to influence the lifespan trajectories of young children's physical activity and health-related physical fitness.

Getting a greater and deeper understanding of children's determinants and correlates of physical activity behaviors and motor skill competence is a multidimensional process involving many cultural, environmental, psychosocial, and behavioral constraints, which may influence individuals' physical activity and fitness trajectories across the lifespan [26–28]. For example, studies that investigated the relationship between physical environment and motor skill competence have yielded inconclusive results, with some showing higher motor skill competence in rural children in comparison with their urban counterparts [29], whereas others reported opposite findings [30]. The discrepancies may be due to the fact that other dimensions, such as social support and perceived competence, are imperative in improving children's motor skill development [31].

Additionally, it is vital to develop novel physical activity promotion programs to foster healthy lifestyle behaviors in young children, which will result in increased physical activity and fitness levels across time with the ultimate goal of promoting health and preventing chronic diseases [5, 6]. As can be seen, investigating the longitudinal sustainability and the impact of novel physical activity interventions on the different dimensions of children's health is needed in future studies, which will help us better understand how innovative physical activity intervention programs can be utilized in communities, homes, and schools to promote a healthy lifestyle throughout the lifespan. Additionally, few studies have examined the longitudinal relationships among young children's physical activity, motor skill competence, screen time, sleep, and other social-ecological variables [32, 33]. Thus, researchers may need to put more emphasis in this area of inquiry, particularly longitudinal research in early childhood. Finally, while the interconnectedness among physical activity, motor skill competence, and cognitive functions have been evident in the past decades [34], it appears that there is a lack of understanding concerning the contextual specificity of these variables [35, 36], which warrants more research on this topic.

*Zan Gao* *Xu Wen* *You Fu* *Jung Eun Lee* *Nan Zeng*
